# Function and Emotion in Everyday Life With Type 1 Diabetes (FEEL-T1D): Protocol for a Fully Remote Intensive Longitudinal Study

**DOI:** 10.2196/30901

**Published:** 2021-10-22

**Authors:** Elizabeth Ann Pyatak, Raymond Hernandez, Loree T Pham, Khatira Mehdiyeva, Stefan Schneider, Anne Peters, Valerie Ruelas, Jill Crandall, Pey-Jiuan Lee, Haomiao Jin, Claire J Hoogendoorn, Gladys Crespo-Ramos, Heidy Mendez-Rodriguez, Mark Harmel, Martha Walker, Sara Serafin-Dokhan, Jeffrey S Gonzalez, Donna Spruijt-Metz

**Affiliations:** 1 Chan Division of Occupational Science and Occupational Therapy University of Southern California Los Angeles, CA United States; 2 Dornsife Center for Economic & Social Research University of Southern California Los Angeles, CA United States; 3 Keck School of Medicine University of Southern California Los Angeles, CA United States; 4 Division of Endocrinology Department of Medicine Albert Einstein College of Medicine Bronx, NY United States; 5 Suzanne Dworak-Peck School of Social Work University of Southern California Los Angeles, CA United States; 6 Ferkauf Graduate School of Psychology Yeshiva University Bronx, CA United States; 7 Department of Psychology University of Southern California Los Angeles, CA United States

**Keywords:** ecological momentary assessments, type 1 diabetes, patient-centered outcomes research, actigraphy, ambulatory monitoring, continuous glucose monitoring, EMA, diabetes, patient-centered outcome, outcome, monitoring, function, emotion, longitudinal, well-being

## Abstract

**Background:**

Although short-term blood glucose levels and variability are thought to underlie diminished function and emotional well-being in people with type 1 diabetes (T1D), these relationships are poorly understood. The Function and Emotion in Everyday Life with T1D (FEEL-T1D) study focuses on investigating these short-term dynamic relationships among blood glucose levels, functional ability, and emotional well-being in adults with T1D.

**Objective:**

The aim of this study is to present the FEEL-T1D study design, methods, and study progress to date, including adaptations necessitated by the COVID-19 pandemic to implement the study fully remotely.

**Methods:**

The FEEL-T1D study will recruit 200 adults with T1D in the age range of 18-75 years. Data collection includes a comprehensive survey battery, along with 14 days of intensive longitudinal data using blinded continuous glucose monitoring, ecological momentary assessments, ambulatory cognitive tasks, and accelerometers. All study procedures are conducted remotely by mailing the study equipment and by using videoconferencing for study visits.

**Results:**

The study received institutional review board approval in January 2019 and was funded in April 2019. Data collection began in June 2020 and is projected to end in December 2021. As of June 2021, after 12 months of recruitment, 124 participants have enrolled in the FEEL-T1D study. Approximately 87.6% (7082/8087) of ecological momentary assessment surveys have been completed with minimal missing data, and 82.0% (82/100) of the participants provided concurrent continuous glucose monitoring data, ecological momentary assessment data, and accelerometer data for at least 10 of the 14 days of data collection.

**Conclusions:**

Thus far, our reconfiguration of the FEEL-T1D protocol to be implemented remotely during the COVID-19 pandemic has been a success. The FEEL-T1D study will elucidate the dynamic relationships among blood glucose levels, emotional well-being, cognitive function, and participation in daily activities. In doing so, it will pave the way for innovative just-in-time interventions and produce actionable insights to facilitate tailoring of diabetes treatments to optimize the function and well-being of individuals with T1D.

**International Registered Report Identifier (IRRID):**

DERR1-10.2196/30901

## Introduction

### Background

Type 1 diabetes (T1D) is an autoimmune disease affecting about 1.6 million people in the United States [1]. T1D is characterized by a near absolute insulin deficiency, requiring intensive management to minimize fluctuations in blood sugar levels. Successfully managing T1D involves consistent ongoing attention to numerous self-care tasks that can be complex and challenging, including monitoring blood glucose levels, taking insulin, managing acute complications, and maintaining supplies and equipment. Such intensive management is needed because blood sugar fluctuations can have a profound impact on everyday life, including swings in emotional states, changes in cognitive functioning, and disruptions to participation in daily activities [2-9]. However, empirical data on these complex relationships within the stream of day-to-day life are limited, as research, to date, has primarily relied on (1) hemoglobin A_1c_ (HbA_1c_) as a measure of blood glucose, which does not capture short-term blood glucose levels and variability [10] and (2) global, retrospective reports of mood, function, and well-being, which do not afford the ability to examine short-term dynamics in subjective experiences and functioning and are often biased by current states and recall problems. A recent review notes a lack of definitive empirical evidence, calling for more rigorous methodology to investigate relationships between glucose variability and mood [11]. This study addresses the call for increased rigor by employing blinded continuous glucose monitoring, accelerometry, ambulatory cognitive tasks, and ecological momentary assessment (EMA) to uncover dynamic associations among blood glucose levels, function, and emotion. Understanding these complex momentary relationships will facilitate tailoring of treatment strategies and development of adaptive, just-in-time interventions to maximize the quality of life among individuals living with T1D.

### Study Aims

**Figure 1:**
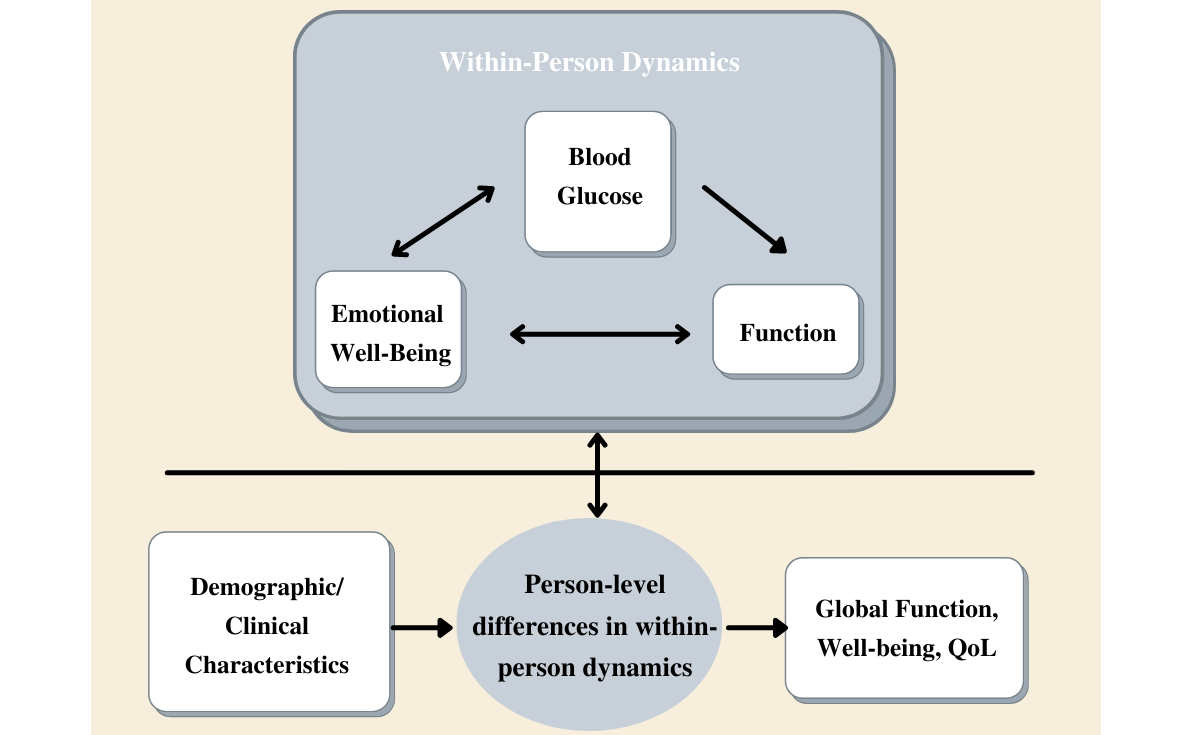
Conceptual diagram of the primary aims of the FEEL-T1D (Function and Emotion in Everyday Life with Type 1 Diabetes) study. QoL: quality of life.

This paper presents the rationale and design of the Function and Emotion in Everyday Life with Type 1 Diabetes (FEEL-T1D) project (NIH/NIDDK #1R01DK121298-01). FEEL-T1D utilizes intensive longitudinal data collection with EMA surveys, ambulatory cognitive testing, and wearable technology (accelerometer, continuous glucose monitor [CGM]) to address 3 primary aims, as depicted in Figure 1. First, we examine within-person dynamic relationships between various measures of blood glucose (acute blood glucose level, glycemic excursions, glycemic variability, time-in-range/hypoglycemia/hyperglycemia), function (self-reported daily life activity performance, objective cognitive function, physical activity derived from accelerometers), and emotional well-being (positive and negative affect, stress, diabetes distress). Second, we evaluate how demographic and clinical characteristics predict individual differences in these within-person effects to inform tailoring of interventions and glycemic targets for population subgroups. Third, we investigate which aspects of these short-term dynamics most impact overall well-being, functioning, and quality of life*.* In doing so, the overall goal of FEEL-T1D is to provide actionable insights for researchers, clinicians, and patients to meaningfully improve health and well-being of people with T1D.

### COVID-19 Impact

The FEEL-T1D study was on the brink of initiating recruitment and data collection in March 2020, when stay-at-home orders in California and New York related to the coronavirus (COVID-19) pandemic required us to reconfigure our planned data collection protocol. Most notably, enforced social distancing practices meant that the planned in-person enrollment, baseline, and follow-up participant visits needed to be conducted fully remotely. Necessary adaptations included maximizing the use of available technology, making use of mailing and delivery services, and selecting measurement tools that were feasible to administer remotely. 

## Methods

### Overview of the Study Design

In the FEEL-T1D project, adults with T1D are asked to complete 14 days of intensive longitudinal data collection using blinded CGM, EMA surveys, ambulatory cognitive tasks, and accelerometer wear. Over 14 days, participants complete 5-6 momentary surveys per day at 3-hour intervals. The first and last surveys of the day ask additional questions to capture information about other constructs on a daily basis. Participants are also asked to complete a baseline survey battery prior to the 14-day period and a follow-up survey battery immediately after the 14 days.

### Participant Recruitment and Eligibility

We are recruiting participants from 3 clinical sites in the greater Los Angeles and New York City metropolitan areas, which collectively serve nearly 2400 ethnically and socioeconomically diverse adults with T1D. Participant eligibility criteria are outlined in Textbox 1. Eligibility criteria were selected to ensure that participants have the ability to complete study procedures and do not have conditions other than diabetes that could significantly influence blood glucose levels. We are seeking to recruit and collect data from a racially, ethnically, and socioeconomically diverse sample to ensure inclusion of underrepresented populations. Furthermore, we aim to enroll participants by using a wide range of diabetes treatment approaches (ie, injections, open-loop insulin pump, closed-loop insulin pump, personal CGM users, and nonusers) to include these regimen differences as potential covariates in analyses. Given the rapidly accelerating uptake of diabetes technologies [12,13] and well-documented differences in clinical outcomes dependent on treatment regimens [14,15], we are eager to investigate whether diabetes technology use has similar implications for mood and functional outcomes. Because starting a new diabetes treatment strategy can influence one’s emotional experiences and take time to develop into a routine, we require that participants be on a stable diabetes therapy for at least 3 months in order to allow time to adjust to the new regimen. For similar reasons, we require that participants taking psychiatric medications be on a stable medication regimen for at least 2 months prior to participation.

**Eligibility criteria for the participants in the FEEL-T1D (Function and Emotion in Everyday Life with Type 1 Diabetes) study.**

**Inclusion criteria**

- Age of 18-75 years (inclusive) at the time of enrollment
- Written and oral proficiency in English or Spanish
- Diagnosis of type 1 diabetes for ≥1 year
- On stable diabetes therapy for >3 months
- >1 month of experience using smartphone (including basic tasks such as texting, emailing, or use of apps)
- Sufficient visual acuity and manual dexterity to manipulate smartphone apps used for data collection
- If on psychiatric medication, on stable medication regimen for >2 months
- Willing and able to complete study procedures
- Participants will be in their normal routine (eg, no unusual or significant events planned during the 2-week data collection period)

**Exclusion criteria**

- Any significant developmental, cognitive, or behavioral conditions (eg, dementia, psychosis) that inhibit completion of study procedures (per observation or medical chart review).
- Currently planning pregnancy, pregnant, or have been breastfeeding for <6 months
- Known adhesive allergy or contact dermatitis that precludes wearing study devices
- Taking systemic corticosteroids (unless on chronic, stable dose at Principal Investigator discretion)
- Planned medical procedure, magnetic resonance imaging, radiography, computed tomography scan, or high-frequency electrical heat (diathermy) treatment during study participation
- Current enrollment in another study that may impact variables assessed in FEEL-T1D
- Currently or within past 14 days has infection or other significant illness (including COVID-19)
- Any other condition that, per study physician review, could interfere with study participation or blood glucose patterns

During the COVID-19 era, the criteria of “experience using a smartphone” and “no illness within the past two weeks” were deemed especially important. Smartphone use was not only evidence that they would be able to follow through with the smartphone (EMA and ambulatory cognitive testing) portion of the study but also an indirect indicator of basic technical ability. Because of the additional technology used in the adapted data collection procedures such as videoconferencing as well as the lack of hands-on training in using the study-provided smartphone, this ability was especially important. In terms of the illness criteria, we were concerned about the possibility of transmitting COVID-19 through incidental exposure during mailing, as well as its impact on participants’ blood glucose levels, mood, and daily activities. Therefore, we decided that any participant who was ill but otherwise eligible for the study needed to be recovered from their illness (irrespective of whether the illness was confirmed to be COVID-19) for at least 2 weeks prior to study participation.

### Recruitment and Retention

Participating sites recruit eligible patients remotely through mailings, phone calls, email invitations, and health provider referrals; previously planned in-person recruitment strategies were eliminated due to COVID-19. Research coordinators have access to patients’ medical charts and contact information at their clinical sites, conduct eligibility screening based on participants’ self-report and medical chart data when relevant and available, and enroll participants over the phone or through videoconferencing. In the case of participants for whom medical chart data is unavailable, eligibility is verified through objective sources (eg, medical records from outside the health system) or through consultation with a study physician prior to study enrollment. The following strategies are being used to maximize retention: (1) daily text messages to provide feedback about survey completion; (2) phone check-ins to resolve questions, address concerns, and provide encouragement; (3) collecting multiple forms of contact information for each participant; and (4) offering graduated stipends where the maximum amount is earned with full completion of the study. Participants earn up to US $200 for completion of all study procedures: US $25 for baseline procedures (disbursed after the baseline call), US $50 for each week that more than 75% of momentary surveys are completed (up to US $100 disbursed after the 2 weeks of data collection), and US $75 for the follow-up procedures and returning the study equipment. In situations where extended data collection is needed owing to reasons such as technical difficulties, additional reimbursement is offered.

### Remote Data Collection Procedures

#### Data Collector Training

Prior to carrying out data collection, research coordinators completed approximately 30 hours of training to master the study procedures and technology. Training materials included video guides and digital manuals. Owing to social distancing requirements, research coordinators needed to become familiar with the technologies that are not part of our previously planned in-person data collection procedures, including videoconferencing software, web-based survey programs, and Google Voice. Additionally, they needed to become accustomed to shipping procedures for study equipment, including disinfecting protocols to minimize the spread of COVID-19. Prior to initiating data collection with participants, research coordinators completed the data collection procedures themselves and conducted a data collection pilot with study team members posing as participants to refine data collection procedures.

#### Screening, Enrollment, and Baseline Data Collection

**Figure 2:**
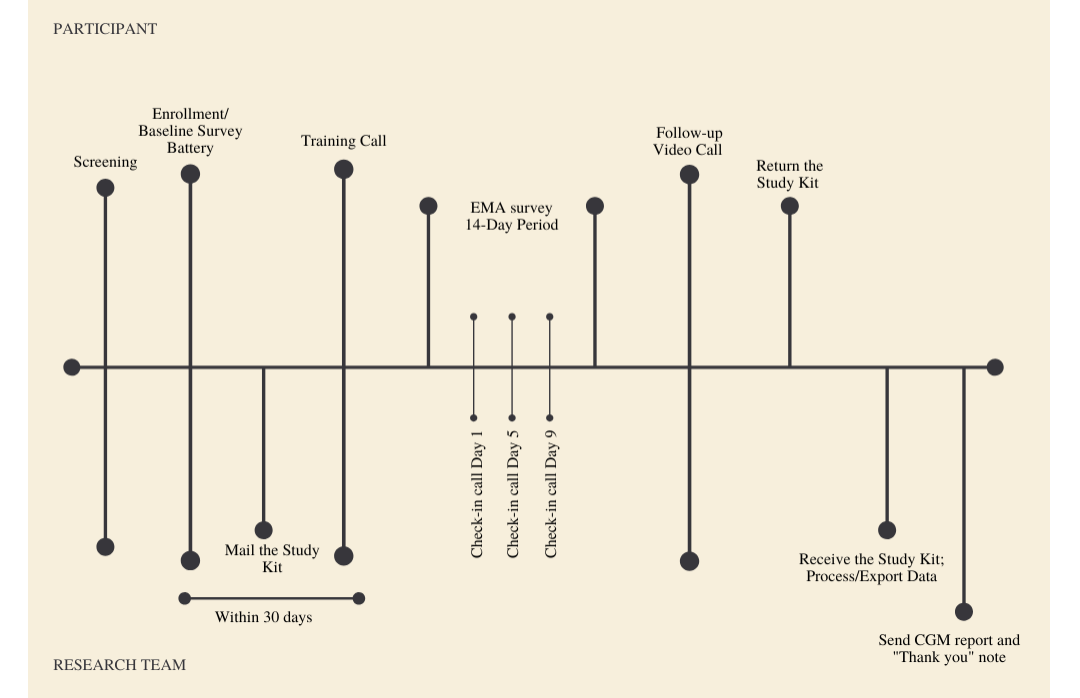
Remote data collection procedures. CGM: continuous glucose monitor; EMA: ecological momentary assessment.

Figure 2 provides an overview of our remote data collection procedures. Participants who are identified as provisionally eligible per medical chart review are contacted by the study team; those who express interest in the study complete a screening questionnaire over the phone. If found to be eligible and interested in the study after the screening, study enrollment can take place. Enrollment paperwork was adjusted to be fully remote. The e-consent framework in research electronic data capture (REDCap), our online data capture platform, is used to record the digital signatures for study enrollment forms, including informed consent, Health Insurance Portability and Accountability Act (HIPAA) authorization, a Loaner Devices Agreement, and Study Stipend form [16]. Lastly, participants complete a baseline survey battery administered via the REDCap survey administration tool.

#### Shipping of the Study Materials

**Figure 3:**
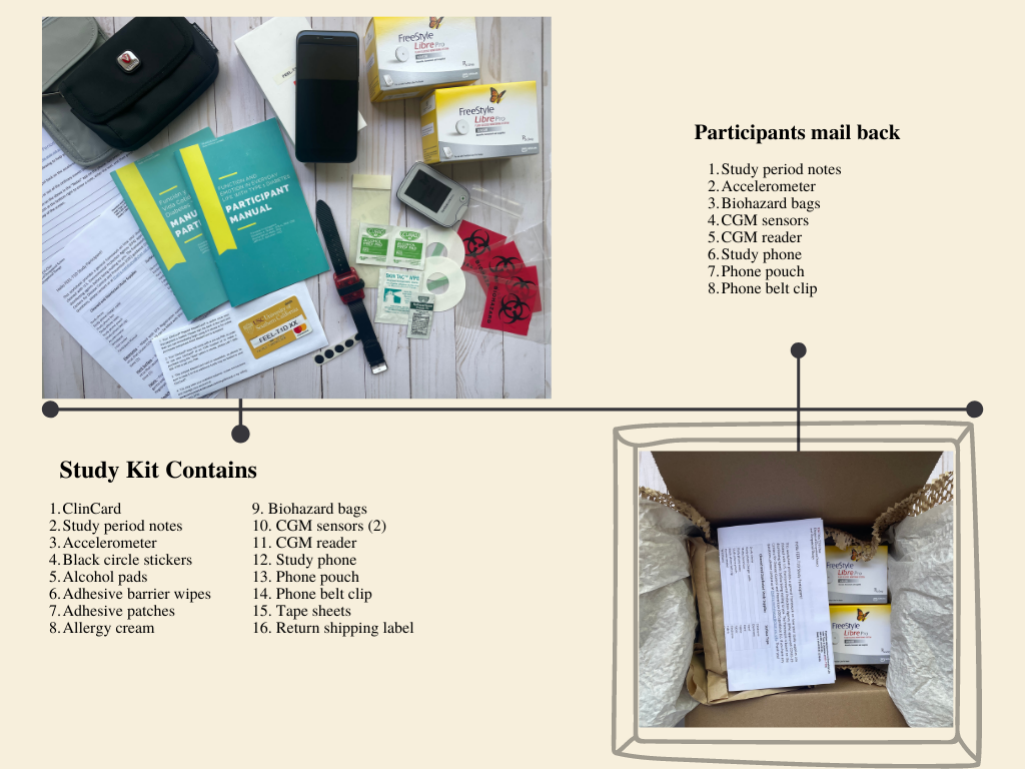
Study materials mailed to the participants. CGM: continuous glucose monitor.

To begin the EMA portion of the study, a box of study materials is shipped to the participants, as shown in Figure 3. These materials include 2 Abbott FreeStyle LibrePro Flash Glucose Monitoring System CGM sensors (a primary sensor and a backup if the first sensor falls off) and a CGM reader (used to activate the sensor; Abbott Diabetes Care), a wrist-worn wGT3X-BT accelerometer (Actigraph), a smartphone (Xiaomi Mi A1) with necessary apps preinstalled and phone accessories, a participant manual, a ClinCard onto which study stipends are loaded, various materials to enhance wearability of devices (eg, adhesive patches, adhesive barrier wipes, allergy relief spray to prevent skin irritation, hydrocortisone cream in the event of an allergic reaction), and materials to return the package after data collection. We are mindful of the possibility that study materials mailed to participants may be lost or damaged and have adjusted our data collection protocol to minimize this risk. Participants are asked to complete baseline surveys before study materials are shipped to them, thereby providing a general indication of their ability and commitment to complete study procedures before sending the materials. Additionally, participants do not receive their final stipend disbursement until all study devices are received in a good working condition, thereby providing a financial incentive to return materials in a timely manner. Finally, study materials are sent with tracking in both directions, higher declared value, fragile shipping labels, and a direct signature requirement, to minimize the possibility of being lost, damaged, or delivered to an incorrect address.

#### Training Call

This call takes place after a participant receives their mailed study materials, using videoconferencing (preferred) or over the phone. The primary purpose of this call is to train participants in use of the study devices. We made efforts to make all the training procedures feasible using only the study phone because not all of our participants have reliable internet access or personal devices to use for videoconferencing (eg, home computer or tablet). Thus, we loaded the necessary videoconferencing software on the study phones and purchased carrier plans providing internet access.

At the beginning of the training call, research coordinators instruct participants on how to self-apply the CGM sensors. This is done first to facilitate checking whether the sensor is operational and recording blood glucose data (which begins after a 1-hour “warm-up” period) before concluding the call. Next, research coordinators guide participants through a participant manual that addresses proper use of all study devices (ie, CGM, accelerometer, study phone), describes the sequence of study events, and explains logistics such as how to mail back the study equipment. Following review of the manual, coordinators guide participants in directed practice with the study phones. To facilitate training over videoconference, participants use the “screen share” feature on the study phone, thereby allowing coordinators to see the participant’s phone screen and to provide instructions accordingly. Participants complete directed practice of all study assessments, during which research coordinators explain each question and response choice to ensure the participant’s understanding, with the phone in “training” mode (in which survey responses are not recorded as study data).

Once the hands-on phone training is completed, usually enough time has elapsed to allow coordinators to check if the CGM is appropriately recording data. If participants are willing to share their insulin pump data, they are asked to prepare the data to be shared at the follow-up visit. If it becomes apparent to research coordinators throughout the training call process that the participant may not have the prerequisite technical skills, cognitive ability, visual acuity, or manual dexterity for successful completion of study procedures, the participant is discontinued from the study.

#### Data Collection

Over the 14 days following the training call, participants complete 5-6 EMA surveys per day at 3-hour intervals over 15 hours (eg, 7 AM-10 PM). The survey schedule is personalized to each participant’s usual weekday and weekend wake and sleep times. If participants have schedules that do not allow for completion of 6 surveys per day, the schedule is adjusted to 5 surveys per day. Participants receive daily text messages providing feedback regarding the previous day’s survey completion and are encouraged to contact the study team whenever any issues occur. Check-in calls, texts, or emails (per participant preference) are conducted at 1, 4, and 8 days after the training call to help ensure continued CGM and accelerometer wear, troubleshoot any technical issues, and encourage completion of surveys. Participants are also encouraged to contact the study team if any issues occur (eg, EMA survey difficulties, CGM falls off).

#### Follow-up Call

A follow-up call is scheduled at the conclusion of the 14 days of data collection. During this call, participants are asked to complete follow-up surveys, answer questions regarding the quality of their experience in using the devices and any unusual events over the 14-day period, and are instructed in how to repackage the study equipment to mail back to the study team with a prepaid return label to return study devices. To minimize burden, we provide the option to schedule a package pick-up from the participants’ homes or other locations.

#### Receiving Returned Equipment

To fully close out a participant, a few steps are taken once the equipment is received. First, all the contents of the package are disinfected in accordance with Centers for Disease Control and Prevention guidelines. Afterwards, contents are checked to make note of any missing equipment. Next, data are downloaded from all the study devices and uploaded to the server, and data loss due to technical issues are noted and communicated to the study team. If we find from the CGM data that a participant spent an excessive amount of time in hypoglycemia (below 54 mg/dL >10% of the time), an alert is triggered and personnel notifies the participant as well as his/her diabetes care provider. When all the study equipment is returned, the participant is provided the final US $75 of the stipend. Additionally, participants receive a thank you letter with a copy of their 2-week CGM report via mail or email if it was requested.

### Study Measures

#### Global Measures

Participants completed 2 survey batteries—one at baseline prior to mailing the study materials (Table 1) and one immediately following the EMA data collection period (Table 2). Participants can elect to complete these surveys on their own or with assistance from a research coordinator, and objective demographic and clinical data are confirmed via medical chart review. Surveys were divided into 2 administration periods to reduce testing burden and because some surveys were intended to reference the period of EMA data collection and thus are administered at follow-up. The purpose of the global measures are to (1) characterize the study population; (2) examine how short-term relationships among blood glucose levels, functions, and well-being differ between patients based on their global demographic and clinical characteristics; and (3) investigate how individual differences in these short-term relationships are related to the global well-being and functioning measures. Overall, in selecting global measures, we prioritized breadth over depth. Although the assessment battery is lengthy, we aimed for parsimony when possible, selecting the shortest validated measure for each construct to maximize the number of assessments that could reasonably be included without inducing undue participant burden.

**Table 1:** Baseline global measures.

Construct			Assessment		Description
**Background variables**					
	N/A^a^	Demographic questionnaire		Gender, ethnicity, education, income, health care coverage, marital status, employment status	
	N/A	Clinical information		Recent severe high/low blood glucose events, method of insulin delivery, pump/injections/continuous glucose monitor use, diagnoses, height and weight	
	Personality	10-item personality inventory [17]		10 items, measures personality along 5 dimensions	
**Diabetes management**					
	Self-management	Diabetes self-management questionnaire [18]		16 items, higher scores indicate more desirable self-management behavior	
	Insulin self-management	Insulin self-management		3 items, inspired by medication adherence items [19], also administered at follow-up	
	Diabetes self-care	Self-Care Inventory-Revised [20]		9 items, higher scores signal increased levels of diabetes self-care, 4 subscales	
**Emotional well-being**					
	Fear of hypoglycemia	Hypoglycemic attitudes and behavior scale [21]		14 items, higher scores indicate more fear of hypoglycemia	
	Anxiety	Generalized Anxiety Disorder Assessment [22]		7 items, higher scores indicate increased severity of anxiety	
	Diabetes stigma	Type 1 Diabetes Stigma Assessment Scale [23]		8 items, higher scores indicate more diabetes stigma experienced, 3 subscales	
	Emotional regulation	Difficulties in emotion regulation scale [24]		18 items, higher scores reflect greater difficulty with emotion regulation	
	Depressive symptoms	Patient health questionnaire [25]		8 items, higher scores reflect greater depression symptoms severity	
**Other**					
	Occupational balance	Occupational balance questionnaire [26]		11 items, higher scores indicate a higher level of lifestyle balance	
	Social support	Social support questionnaire [27]		12 items, higher scores signal greater satisfaction with social support system	

^a^N/A: not applicable.

**Table 2:** Follow-up global measures.

Construct		Assessment	Description
**Function**			
	Functional health status	RAND 36-item short form health survey v1.0 [28]	36 items, measures 8 dimensions of health, higher scores indicate better functional health status
	Illness intrusiveness	Adapted illness intrusiveness rating scale [29]	13 items, higher scores reflect greater interference associated with the disease (diabetes) and its treatment
**Emotional well-being**			
	Diabetes-related quality of life	Helmsley quality of life and diabetes survey [30]	27-36 items depending on age group, higher scores reflect better diabetes-related quality of life
	Diabetes distress	Problem areas in diabetes scale [31]	5 items, higher scores suggest greater diabetes-related emotional distress
	Positive and negative affect	Stress and Working Memory Study Affect Items [32]	9 items, sum of 4 items indicates positive affect and sum of other 4 items indicates negative affect; 1 item not from original (“tension”) was added
	Perceived stress	Perceived stress scale [33]	10 items, higher scores indicate greater perceived stress
	Life satisfaction	Satisfaction with life scale [34]	5 items, higher scores reflect greater life satisfaction
**Other**			
	N/A^a^	COVID-19 questions	Provides information about COVID-19 era life circumstances such as economic and lifestyle changes
	N/A	Study-specific follow-up questions	Difficulties with the study devices, experience of diabetic ketoacidosis or hypoglycemia during study visit

^a^N/A: not applicable.

Our global assessment battery was adapted to fit the needs of remote research during the COVID-19 pandemic. We dropped 3 planned measures that were not critical to accomplish the study’s aims owing to logistical challenges. One change was eliminating the measurement of HbA_1c_ levels, which capture average blood glucose levels over an approximately 12-week period; the team instead recorded HbA_1c_ readings from the previous 12 months from medical charts, when available, to gain insight into participants’ overall glycemic control as a potential moderator of observed relationships. Additionally, measurements of height, weight, and neck circumference (to assess sleep apnea risk) were dropped, with height and weight now being assessed through self-report. We also removed the National Institutes of Health (NIH) Toolbox cognitive tests [35], which are completed with an in-person test administrator using iPads. Their purpose was to help validate the mobile cognitive assessments being used, but as some validation data already exist for the mobile cognitive assessments, these tests were determined not to be critical. Finally, we added a COVID-19 questionnaire adapted from prior COVID-19 surveys [36,37] to capture the impact of COVID-19–related life changes and help us understand how our study population may be unique as compared to studies conducted before or after COVID-19.

#### EMA Measures

The EMA questions are outlined in brief in Textbox 2. Multimedia Appendix 1 lists the actual items and response options used. EMA data collection is administered using the mobile EMA (Ilumivu) platform; an HIPAA-compliant EMA system, which incorporates a native smartphone app; a web interface for survey design and deployment; and a secure cloud-based server for data management [38].

**Ecological momentary assessment survey questions.**

**Morning questions**

Domains: sleep quality, sleep/wake time, anticipated busyness, diabetes self-efficacy

Questions are only asked in the first survey of every day

**Activity engagement (in all surveys)**

Domains: activity type, activity location, activity social situation, activity performance, activity satisfaction, activity importance, diabetes intrusiveness

Questions about activity performance, satisfaction, and importance were derived from the Canadian Occupational Performance Measure [39]

Question about diabetes interference derived from Adapted Illness Intrusiveness Rating Scale [29]

**Emotional well-being (in all surveys)**

Domains: mood, stress, diabetes distress, fatigue, pain

Mood question formatting was derived from the Positive and Negative Affect Schedule [40], and actual items were from the Stress and Working Memory [32]

**Blood glucose (some parts in all surveys)**

Domains: Meal intake (yes/no), meals time, insulin intake (yes/no), insulin time, perception of blood glucose

Items derived from a prior diabetes ecological momentary assessment study [41]

Questions referencing the last 3 hours are asked in all surveys except the first survey of every day

**Evening questions**

Domains: activity performance, insulin self-management, diabetes self-management, study devices statuses, unexpected events, perceived daily demands

Daily demand questions were adapted from the National Aeronautics and Space Administration–Task Load Index [42]

Questions asked in last ecological momentary assessment survey of every day

#### Momentary Surveys

Survey questions were selected on the basis of being derived from validated global measures or having been used successfully in previous EMA studies [29,39,41-45]. Participants answer approximately 30 survey items in the first 5 surveys of the day and 50 items in the evening survey (depending on branching logic).

#### Ambulatory Cognitive Assessments

Cognitive performance is assessed with 2 tests taken 6 times daily on the study phone. A “Go/No-Go” task assesses inhibitory control [46] and consists of 75 trials that take approximately 1 minute. Participants are presented a series of images that are either mountains or cities. They are asked to press a button when they see an image of a city but refrain from pressing the button if images of mountains are presented. A “Symbol Search” task assesses visual-spatial attention and processing speed [47] and consists of 20 trials that take approximately 45 seconds. Participants are presented with 2 cards at the top of the screen and 2 at the bottom. They are asked to choose a card from the bottom of the screen that matches one of the cards from the top. Cognitive tests administered through phones have been found to be valid as evidenced by demonstrating expected associations with measures of cognitive testing delivered through in-lab assessments [47]. Retest gains (training effects) are common when cognitive tests are repeated multiple times. Participants in this study undergo careful training of the study procedures and complete the cognitive tests for the first time as part of the training session, and these scores do not enter the analyses. Even though our test stimuli are unchanged across assessments, this may mitigate retest effects to some extent. To evaluate the robustness of results to potential retest gains, we will conduct sensitivity analyses in which the first few cognitive scores are removed from the analyses, and we will examine detrended cognitive scores where individual trends in test scores due to retest effects (eg, exponential decay of response times) are statistically removed from the data.

### Study Devices

One of our primary study devices is Abbott’s Freestyle Libre Pro Flash Glucose Monitoring System CGM. To ensure consistency, all participants wear this CGM, regardless of whether they also wear a personal CGM. After initial placement on the back of the upper portion of the participants’ arms, it automatically records glucose levels from interstitial fluid (which is converted via an algorithm to estimate the blood glucose levels) at 15-minute intervals continuously for 2 weeks. CGM data are processed by Abbott using the Freestyle Libre2 Flash Glucose Monitoring System algorithm that meets integrated CGM performance requirements because this algorithm is not yet integrated in the Libre Pro CGM.

The Actigraph wGT3X_BT wrist accelerometer was another core study device. It provides continuous data that can be used to infer time spent in sedentary, light, moderate, vigorous physical activity, and sleep each day [48]. To better account for possible errors in sleep measurement with the Actigraph alone, sleep/wake times are calculated using both Actigraph data and self-reports of sleep/wake times through a weighted average approach as recommended in prior research [49].

Finally, the study phones used were Xiaomi Mi A1 models with Android operating systems. They were chosen because they were relatively inexpensive, had sufficient processing power and screen size to run the cognitive tests, and because the Android operating system was preferred by EMA and cognitive testing programmers. Participants were given study phones rather than using their own devices primarily to ensure the comparability of cognitive testing results. If participants used their own devices, there was a possibility that factors such as differences in the phone processing speed or screen size could affect the cognitive testing scores.

### Analytic Plan

Standard statistical diagnosis and descriptive statistics will be used to evaluate the reasonableness, sparseness, and potential nonnormality of the data. Psychometric properties of EMA multi-item scales (eg, mood) will be investigated, including multilevel factor analysis, to confirm the dimensionality of self-report measures, cross-level invariance, and adequate internal consistency of scale scores in between-person and within-person levels [50,51]. Univariate analyses of temporal patterns will be used for some variables to examine diurnal rhythms and systematic trends over time. We will check for outliers and investigate their potential causes, including technical glitches (eg, surveys being delivered at unanticipated times due to time zone changes) and satisficing (putting minimal effort into survey or responding to finish quickly).

Data analysis will be conducted using Dynamic Structural Equation Modeling (DSEM). This method combines multilevel modeling and time-series analysis into a unified framework, allowing for the analysis of multivariate time series obtained from multiple individuals simultaneously [52-55]. Multilevel modeling is a form of linear regression that accounts for nested data (multiple observations nested in individuals) [56]. Rather than analyzing a time series model separately for each individual, DSEM enables us to examine the magnitude and directionality of dynamic relationships between blood glucose and other measures within individuals, while simultaneously allowing for the analysis of quantitative differences in these relationships across individuals in the same model. Larger sample sizes (N=200 in this study) can compensate for shorter time series [55].

Aim 1 focuses on assessing the within-person relationships between blood glucose measures, function, and emotional well-being. Analyses for this aim will begin by checking for between-person and within-person correlations between blood glucose and functioning/emotional well-being variables to gauge the relative magnitude of within-person versus between-person correlations. Lagged temporal relationships between blood glucose and other momentary variables will be examined with DSEM. For instance, DSEM would allow us to elucidate if negative affect precedes hyperglycemia, hyperglycemia precedes negative affect, or if their relationship is bidirectional within or across days.

In aim 2, possible moderators (eg, sex, race/ethnicity, CGM use) of the observed within-person relationships among blood glucose measures, function, and emotional well-being are assessed. For example, we will investigate whether personal CGM use moderates relationships between blood glucose measures and well-being. Because CGM users likely have much greater awareness of their blood glucose levels, we anticipate that their cognitive evaluation of blood glucose may impact their mood, in addition to any physiological pathways between blood glucose measures and mood. As another example, we will examine whether prolonged nocturnal hypoglycemia moderates relationships between blood glucose levels and momentary cognitive functioning. To test potential moderators, cross-level (person-by-situation) interactions will be examined using traditional multilevel modeling and (for more complex models involving moderators of effects in multivariate analyses) DSEM.

For aim 3, we investigate how individual differences in momentary (within-person) associations between blood glucose levels and functioning/well-being relate to global measures of functioning, well-being, and quality of life. For example, patients whose momentary cognitive functioning is more strongly affected by their momentary blood glucose levels may show worse functioning levels overall than patients whose cognitive functioning remains relatively unaffected by fluctuations in their blood glucose levels. If significant effects are found in these analyses, we will also explore the possibility that individual differences in the dynamic relationships between blood glucose and other momentary measures *mediate* the relationships between demographic/clinical characteristics and global measures of functioning and well-being. A hypothetical example would be that men and women differ in how momentary blood glucose affects their momentary mood and that this in turn explains gender differences in the overall mental well-being. DSEM methods will again be used here, where global functioning measures will serve as dependent variables, and random effects (ie, latent individual differences) of the short-term (within-person) dynamics will serve as independent variables or as intermediate variables (in potential mediator models) [53].

Prior to collecting and analyzing the full data set, we are investigating simpler subquestions of our overarching aims to help determine how to best model the data. One “simple” question, for instance, is how high blood glucose levels affect functioning at the within-person level (aim 1). This seemingly innocuous question brings with it a host of other queries: *What parameters of a high blood glucose level need to be considered (eg, time with high blood glucose levels, time since high blood glucose levels were detected, magnitude of high blood glucose levels)?* and *What variables best capture functioning: self-report or objective cognitive measures?* Another subquestion is how nighttime blood glucose affects functioning the next day, which requires consideration of *How should night-time blood glucose be summarized (eg, average blood glucose levels, time in range, coefficient of variation, etc?)* and *How can we delineate the time sleeping given our available data?* As we tackle these subquestions, we will gradually increase our understanding of the data and the best fitting models to be better prepared to conduct more robust analyses when the full data set is collected. This process is necessary, given the novelty of analyzing within-person momentary relationships among blood glucose levels, functioning, and emotion.

### Sample Size and Power Considerations

We designed the study to have at least 80% power for the detection of the anticipated effect sizes of .10 (3% of the variance) to .25 (6% of the variance), corresponding with small to medium effects. Power calculations were conducted using Monte Carlo simulations, assuming a ratio of random intercept to within-person residual variance of 1.5/1, a ratio of random intercept to random slope variance of 5/1, a first-order autocorrelation of 0.4, and 80% compliance with EMA (based on our prior research). A sample size of 200 patients observed 6 times/day over 14 days will provide 80% power (α=.01, adjusted for multiple comparisons) to detect an effect size of .10 for lagged within-person relationships (aim 1), an effect size of .18 for cross-level interactions (moderators of within-person relationships, aim 2), and an effect size of .23 for random effects of within-person relationships as predictors of between-person outcomes (aim 3).

## Results

Since initiating data collection in June 2020, our goal has been to recruit approximately 11 participants per month to attain our targeted sample size of 200 participants within 18 months. Following 12 months of recruitment, 124 participants have successfully enrolled in the FEEL-T1D study (excluding 4 patients who did not complete baseline assessment), and we project to complete enrollment by November 2021 (Table 3). Overall, remote study implementation has been a success. Weekly meetings are held to discuss study implementation issues that arise, and team members who are experts on various aspects of data collection are consulted as needed. The details of the study implementation to date are given in Table 3.

**Table 3:** Study implementation statistics (as of May 31, 2021).

Statistics		All sites, n (%)	Westside Center for Diabetes, n (%)	Los Angeles Roybal Clinic, n (%)	Einstein College of Medicine/Montefiore Medical Center, n (%)
Participants enrolled		124 (100.0)	37 (29.8)	36 (29.0)	51 (41.1)
Participants withdrew		5 (4.0)	3 (8.1)	2 (5.5)	0 (0.0)
Participants in progress		19 (15.3)	5 (13.5)	9 (25.0)	5 (9.8)
Participants completed		100 (80.6)	29 (78.4)	25 (69.4)	46 (90.2)
**Data quality: number of days with concurrent ecological momentary assessment, continuous glucose monitor, and accelerometer data**					
	10 days or more	82 (82.0)	23 (79)	20 (80)	39 (85)
	1-9 days	11 (11.0)	1 (3)	5 (20)	5 (11)
	0 days	7 (7.0)	5 (17)	0 (0)	2 (4)

Overall, adherence and data quality have been very good thus far (Table 4). The EMA survey compliance rate has been consistently high (7082/8087, 87.6% of all prompts) as has completion of ambulatory cognitive assessments (6795/8087, 84.0% compliance). Of the 100 participants who completed the study at the time of this report, 82 participants provided concurrent CGM, EMA, and accelerometer data for at least 10 of the 14 days of data collection. Of those who did not provide complete data, problems included incomplete (<7 days) or missing CGM data (n=6 and n=5, respectively) and incomplete (<7 days) or missing accelerometer data (n=8 and n=6, respectively), with some participants having missing data from both devices. Five participants who enrolled did not complete the study: reasons included personal/family emergency (n=1), feeling that the study was too burdensome (n=2), and acute health conditions (n=2).

**Table 4:** Data of survey completion.

Survey completion data	Overall (N=8087)	Morning (n=1400)	Midday (n=5287)	Evening (n=1400)
Surveys completed, n (%)	7082 (87.6)	1232 (88.0)	4629 (87.6)	1221 (87.2)
Duration (min) (excluding cognitive tests), mean (SD)	2.9 (3.1)	3 (2.8)	2.4 (3.1)	4.5 (2.8)

## Discussion

FEEL-T1D is, to our knowledge, the first study to examine dynamic reciprocal relationships between blood glucose levels, objective cognitive and physical function, and subjective function and well-being among adults with T1D. It is one of only 3 studies, to our knowledge, using EMA methodology to investigate associations between CGM-derived blood glucose metrics and other momentary variables among individuals with diabetes, with the other 2 being the international HypoRESOLVE (Hypoglycemia Refining Solutions for Better Lives) project [57] and the DIA-LINK study in Germany [58]. This study is innovative in its use of CGM, EMA, mobile cognitive testing, and accelerometry, analyzed with sophisticated statistical methods, to achieve its aims. Knowledge generated from this study will provide actionable insights for researchers, clinicians, and people living with diabetes by facilitating tailoring of diabetes treatments to maximize function and well-being in addition to physical health and by informing the development of interventions that address the dynamic relationships between these constructs. Furthermore, to increase the generalizability of our results, we are recruiting a diverse sample with respect to race, ethnicity, socioeconomic status, and diabetes treatment approaches. We attribute our success in the study’s implementation, to date, to a carefully crafted set of procedures for remote data collection to promote participant adherence to the study protocol, abide by social distancing requirements necessitated by COVID-19, and maintain the quality of data collected.

Fully remote implementation comes with several benefits. Foremost among them is that, owing to restrictions on in-person contact and our ethical obligation to protect study participants from harm, conducting the study during the COVID-19 era *would not have been possible* without remote implementation. When California and New York issued stay-at-home orders in March 2020, with an uncertain future ahead, our choices were to adapt our study procedures or to wait indefinitely until in-person data collection was feasible again. Additionally, remote data collection has also allowed us to enroll participants living far away from study sites. Thus, the creation of a remote protocol has increased recruitment opportunities and potentially diversified our study population. Remote data collection has also freed us from logistical challenges related to in-person data collection, such as ensuring that participants have access to parking and transportation when visiting study sites. Relatedly, scheduling study appointments has been much easier because participants can complete them at home instead of having to factor in transportation costs and time. Finally, remote data collection has given us the option to more neatly enact division of labor, thereby enabling team members to specialize and perform study procedures more efficiently. Some team members specialize in recruitment and participant contacts, while others focus on preparing and shipping data collection kits or processing data. In our previously planned in-person arrangement, each research coordinator was responsible for a wide range of tasks, including walking participants through data collection, setting up devices to be loaned to participants, and downloading data from devices when returned. Because of the logistical complexity of the various tasks, allowing team members to specialize has been more efficient.

Despite its benefits, the transition to remote data collection has also had some drawbacks. We were unable to use some of the measures we had initially planned on administering, such as the NIH toolbox and point-of-care HbA_1c_, and therefore cannot compare our mobile cognitive testing and continuous blood glucose data to their more traditional counterparts. Another drawback has been the additional requirement for our research coordinators and study participants to be adept in using videoconferencing software. Because our less technologically savvy participants are disproportionately older or live in areas with poor internet connectivity, the generalizability of our study results among these populations may be limited. In addition, inherent in fully remote study procedures has been a greater frequency of technical and mailing issues. Unexpected software updates have caused videoconferencing not to work, and internet connection issues have led to the postponement of scheduled video calls. Unforeseen mailing delays have led to delays in study appointments, and misplaced packages require extra effort to track down. Finally, shipping and processing our data collection equipment is costly and has required significant personnel time.

Assuming that the effects of COVID-19 will continue over the duration of our data collection, we will not have a way to compare COVID-19 and non-COVID-19 pandemic participants. With data gathered from the FEEL-T1D COVID-19 questionnaire that addresses life changes as a result of the pandemic, we may be able to provide descriptive data characterizing our sample and thereby allowing basic comparison to “normal” participants. Questions asked include “Compared to before the coronavirus outbreak, how would you now describe your current overall level of stress or worry?” and “Have you experienced any of the following major life changes related to the coronavirus outbreak?” (eg, laid off or furloughed, having children at home who usually attend school, camp, or daycare, and major change in the health of a family member).

In summary, the FEEL-T1D study aims to fill gaps in the knowledge about the relationships between short-term blood glucose levels and both momentary functioning and well-being. Our efforts to launch the study were delayed by the COVID-19 pandemic, but we were able to reconfigure our data collection protocol to be fully remote. With our reconfigured procedures, we have successfully recruited participants and have high completion rates over 2 weeks of nontrivial data collection, in spite of the challenges of conducting research with social distancing requirements in effect. We anticipate that the data provided by the FEEL-T1D study will answer questions of importance to the T1D community regarding optimal glycemic patterns for mood changes and functional ability and facilitate individualized tailoring of treatments to maximize the well-being and quality of life of persons with T1D.

## Appendix

Multimedia Appendix 1 Full ecological momentary assessment survey battery.

## Abbreviations

CGM: continuous glucose monitor
DSEM: dynamic structural equation modeling
EMA: ecological momentary assessment
FEEL-T1D: Function and Emotion in Everyday Life with Type 1 Diabetes
HbA_1c_: hemoglobin A_1c_
HIPAA: Health Insurance Portability and Accountability Act
NIH: National Institutes of Health
REDCap: research electronic data capture
T1D: type 1 diabetes
