# Supplementary material for: Function and Emotion in Everyday Life With Type 1 Diabetes (FEEL-T1D): Protocol for a Fully Remote Intensive Longitudinal Study
Source: JMIR Res Protoc. 2021 Oct 22;10(10):e30901. doi: 10.2196/30901 (PMC8544739; doi:10.2196/30901)
Supplement: Multimedia Appendix 1 [file resprot_v10i10e30901_app1.docx]

**Appendix 1. Full EMA Survey Battery**

| **General Category and Descriptions** | **Domain** | **Item/Description** | **Response Options** |
| --- | --- | --- | --- |
| MORNING QUESTIONS  - Questions are only asked in the first survey of every day | Sleep quality | How well rested do you feel? | 0 (Not at all rested) to 100 (Extremely rested) |
|  | Sleep/wake time | What time did you go to bed/wake up? | HH:MM |
|  | Anticipated busyness | How busy will your day be? | 0 (Not at all busy) to 100 (Very busy) |
|  | Diabetes self-efficacy | How confident do you feel about managing your diabetes today? | 0 (Not at all confident) to 100 (Very confident) |
| ACTIVITY ENGAGE- MENT  - Questions about activity performance, satisfaction, and importance were derived from the COPM [44]  - Question about diabetes interference derived from A-IIRS [48]  - Items asked in all surveys | Activity type | What were you doing right before starting this survey? | Work/school, traveling, relaxing/chilling, sleeping/napping, socializing, caring for myself, caring for others, doing housework/ errands, fun/play/leisure activities, other |
|  | Activity Location | Where were you when doing this activity? | At home, at work outside the home, school outside the home, outdoors, other |
|  | Activity Social Situation | Who were you with when doing this activity? | No one, spouse/partner, co-worker(s)/classmate(s), friend(s), my child/children, other family members, pets, other |
|  | Activity Performance | How well were you able to do this activity? | 0 (Unable to do it at all) to 100 (Did it extremely well) |
|  | Activity Satisfaction | How satisfied are you with the way you did this activity? | 0 (Not satisfied at all) to 100 (Extremely satisfied) |
|  | Activity Importance | How important is this activity to you? | 0 (Not important at all) to 100 (Extremely important) |
|  | Diabetes Intrusiveness | Did your diabetes get in the way of doing this activity? | No, Yes because of how I was feeling, Yes because of my device(s), Yes because I was taking care of my diabetes |
| EMOTIONAL WELL- BEING  - Mood question formatting was derived from the PANAS [49], and actual items were from the SAWM [35]  - Items asked in all surveys | Mood | Right now, how _ do you feel? (happy, content, enthusiastic, excited, tense, upset, sad, disappointed, anxious) | 0 (not at all) to 100 (extremely) |
|  | Stress | How stressed are you right now? | 0 (Not at all stressed) to 100 (Extremely stressed) |
|  | Diabetes Distress | How stressed do you feel about your diabetes or diabetes management right now? | 0 (Not at all stressed) to 100 (Extremely stressed) |
|  | Fatigue | At this moment, how tired do you feel? | 0 (Not at all) to 100 (Extremely) |
|  | Pain | At this moment, how much bodily pain do you have? | 0 (None) to 100 (Extreme pain) |
| BLOOD GLUCOSE (BG)  - Questions referencing the last 3 hours were asked in all surveys except the first survey of every day  - Items derived from a prior diabetes EMA study [43] | Meals/Insulin Intake | Did you eat/drink/take insulin eat or drink in the last 3 hours? | I ate, I drank, neither |
|  | Meals Time | (If ate or drank) When did you eat/ drink? Check all that apply | less than an hour ago, 1 to 2 hours ago, 2-3 hours ago |
|  | Insulin | Have you taken any insulin in the last 3 hours? | Yes/No, (If yes) injection or pump |
|  | Insulin Time | When did you take insulin? | less than an hour ago, 1 to 2 hours ago, 2-3 hours ago |
|  | Perception of BG | How do you think your blood sugar changed in the last 3 hours? | Went up, down, went up and down, no change |
|  | Perception of BG | How does your blood sugar feel right now? | very low, low, just right, high, very high |
| EVENING QUESTIONS  - Daily demands questions were adapted from the NASA-TLX [42]  - Questions asked in last EMA survey of every day | Act. Performance | Were there activities you could not do because of diabetes? | Check off activities from “activity type” question above |
|  | Act. Performance | Were there activities you had to do differently because of diabetes? | Same as above |
|  | Insulin self- management | Today, how did you do at taking your insulin? | 0 (Very Poor) to 100 (Excellent) |
|  | Diabetes self- management | Today, how did you do at your overall diabetes self-care? | 0 (Very Poor) to 100 (Excellent) |
|  | Study devices status | Did you take off the accelerometer today?/ Is the CGM securely attached? | Yes/No |
|  | Unexpected events | Did anything out of the ordinary happen today? | Yes/No |
|  | Perceived daily demands | Questions about perceived demand in the following dimensions: mental, physical, time pressure, effort, performance, frustration level | 0 to 100 sliding scale for each item |
